# On Top of Everything: a study protocol for a cluster-randomised controlled trial testing a teacher training programme to teach mindfulness among students in Danish upper secondary schools and schools of health and social care

**DOI:** 10.1186/s13063-022-06920-7

**Published:** 2023-01-06

**Authors:** Michelle Sand Beck, Lise Juul, Morten Frydenberg, Lone Overby Fjorback

**Affiliations:** 1 Department of Clinical Medicine, Danish Center for Mindfulness, Aarhus University, Hack Kampmanns Plads 1-3, 4Th Floor, 8000 Aarhus C, Denmark; 2 MFStat, Aarhus, Høgemosevej 19A, 8380 Trige, Denmark

**Keywords:** Pragmatic clinical trial, Mental health, School-based intervention, Mindfulness-based stress reduction, MBSR, Mindfulness, Adolescent, Young adult

## Abstract

**Background:**

Mental health is decreasing among young people in Denmark. Our primary aim is to evaluate the effectiveness of a teacher training programme to teach mindfulness as part of regular classroom teaching in Danish upper secondary schools and schools of health and social care on students’ self-reported mental well-being 6 months from baseline. Secondary aims are (1) to evaluate the effectiveness in a vulnerable subgroup as well as in the total population of students 3 and 6 months from baseline using other outcome measures on mental health and (2) to investigate the facilitators and barriers among teachers to implement mindfulness in schools.

**Methods:**

This pragmatic cluster-randomised two-arm superiority trial includes 30 upper secondary schools, 13 schools of health and social care, 76 teachers, and approximately 1100 students aged 16 to 24 years. Our intervention is multi-level and consists of (a) a teacher training programme and (b) a mindfulness programme delivered to students. Students in control schools receive education as usual. Our primary study population is the total population of students. The primary outcome is changes in the short version of the Warwick-Edinburgh Mental Well-Being Scale (SWEMWBS). We also evaluate the effectiveness in a vulnerable subgroup (the 15% with the lowest SWEMWBS score), as well as in the total population of students 3 and 6 months from baseline using other outcome measures on mental health. Data will be analysed using repeated measurement models taking clusters into account. Facilitators and barriers among teachers to implement mindfulness in schools will be investigated through qualitative focus group interviews.

**Discussion:**

The trial will estimate the effectiveness of a population-based strategy on mental health in Danish young people enrolled in education.

**Trial registration:**

ClinicalTrials.gov NCT04610333. Registered on October 10 2020.

## Background

Mental health of children and young people is a global public health challenge [1]. The need to focus on the mental health of young people is gaining increasing recognition as the global community looks to achieve the ambitious Sustainable Developmental Goals (SDG), such as SDG 3: “Ensure healthy lives and promote well-being for all at all ages” [2]. Mental health conditions account for a considerable proportion of the global disease burden during youth and are the leading course of disability in young people [3]. In fact, 75% of mental disorders begin before the age of 24 and 50% by the age of 15 [4]. Suicide is one of the three leading causes of death among older adolescents [5]. Therefore, mental health conditions have huge costs for the individuals affected as well as for society as a whole. The total costs related to mental health problems in Europe are estimated to account for more than EUR 600 billion. Among the European countries, Denmark has the highest costs in terms of % of GDP (5.4%) [6].

However, mental health is more than the absence of mental disorders [7]. Mental health is a fundamental component of the World Health Organization’s (WHO) definition of health. It is conceptualised as a state of well-being in which the individual realises her or his own abilities, is able to cope with the normal stresses of life, can work productively and fruitfully, and is able to make a contribution to her or his community [8]. In this positive sense, mental health is the foundation for well-being and effective functioning for an individual and a community [7].

In Denmark, mental health among young people has been decreasing in recent years. Since 2010, the National Health Profile has reported an increase in the proportion of 16–24-year-olds with poor mental health [9]. A high occurrence of stress, anxiety, tension, and loneliness is seen in this age group as well [10].

According to WHO, mental health is a public issue that requires political priority and action. This is to ensure that effective promotion and prevention are established and that stigmatisation and discrimination are broken down [11]. Mental health promotion and disease prevention in young people can benefit young lives in the short and long term. This stage is deemed as one of the optimal timeframes for intervention, given the neuroplasticity evident in adolescence and the opportunity to step in at a time when the majority of mental health conditions and risky behaviours have their onset [5]. WHO recommends population-based psychosocial interventions to promote positive mental health and prevent mental disorders [3]. Likewise, The Lancet Commission on global mental health and sustainable development emphasise the importance of improving mental health for whole populations [12]. Population-based interventions have the highest and most valuable impact at a society level [13]. The reason for this is that most cases of poor mental health occur among the many who are at low or moderate risk, rather than among the few who are at high risk. In addition, population-based interventions have the potential to improve mental health in all young people as well as in high-risk and vulnerable groups without causing stigma [13].

School environment has been identified as an appropriate setting for providing mental health promotion and prevention. Not least because of its broad reach and central role in the lives of young people [14, 15]. O’Conner highlights the importance of providing school teachers with the necessary skills and knowledge to ensure that the school setting is a beneficial environment for promoting mental health [14]. However, there is a lack of evidence-based intervention content. Moreover, many strategies to improve mental health are designed to be used when people are unwell, and therefore lack relevance for those at low risk and for those who are at high risk, but not currently showing symptoms [16].

Since life cannot be lived without challenges and discomfort, training the ability to handle and be with these challenges in an appropriate way is necessary and beneficial for everyone. One way to train this is through the practice of mindfulness. Mindfulness is a method of mental health training that can be used by anyone, regardless of how they feel. Mindfulness has been defined as the awareness that arises from paying attention on purpose in the present moment, non-judgementally, in the service of self-understanding, wisdom, and compassion [17]. Mindfulness-based Stress Reduction (MBSR) is a well-described group-based programme in mindfulness training developed in 1979 by Jon Kabat-Zinn [18]. The programme consists of eight weekly sessions of 2 ½ hours and one all-day session. The sessions have standardised core elements consisting of different mental and psychical mindfulness exercises. Additionally, there are teachings on stress, stress management, and how to apply mindfulness to interpersonal communication and everyday situations [18]. Today, MBSR is an incorporated part of both mental health promotion, prevention, and treatment worldwide. A Campbell Systematic Review based on 101 randomised controlled trials concludes that MBSR has a moderately large positive effect on outcome measures of mental health when compared to inactive controls. The intervention also improves the quality of life, including social function, and personal development like empathy, coping, sense of coherence, and mindfulness. Compared to active controls, MBSR has an additional small positive effect on outcome measures of mental health, depression, stress, and mindfulness [19].

Given the beneficial effects established by MBSR in adults, there has been an increasing interest in implementing similar initiatives for young people. Research in this area is still preliminary, but growing. Dunning et al. conclude the positive effects of mindfulness-based interventions on mental health in children and adolescents [20]. Their meta-analysis is based on 33 randomised controlled trials. However, there is a high degree of variability in intervention contents and target groups, and the meta-analysis emphasises the importance of incorporating scaled-up trials to further evaluate the robustness of mindfulness-based interventions in youth [20].

The Danish Parliament has funded the Danish Center for Mindfulness at Aarhus University to educate upper secondary school teachers and teachers at schools of health and social care to teach mindfulness to students. The Danish Center for Mindfulness has systematically developed a version of MBSR modified for young people called “On Top of Everything”. It is built on the same overall themes and mindfulness exercises as MBSR, but the duration, number of sessions, and focus in the exercises are adapted to young people in a classroom setting.

## Aims

Our primary aim is to evaluate the effectiveness of a teacher training programme to teach the mindfulness programme “On Top of Everything” as part of regular classroom teaching in Danish upper secondary schools and schools of health and social care on students’ self-reported mental well-being 6 months from baseline. Secondary aims are (1) to evaluate the effectiveness in a vulnerable subgroup as well as in the total population of students 3 and 6 months from baseline using other outcome measures on mental health and (2) to investigate the facilitators and barriers among teachers to implement mindfulness in schools.

## Hypothesis

We hypnotise that young people in the intervention group will improve their mental health during the intervention. We expect to find small effect sizes in the total population of students and higher effect sizes in a vulnerable subgroup [13].

## Methods/design

### Design

The study is designed as a pragmatic cluster-randomised two-arm superiority trial including 30 upper secondary schools and 13 schools of health and social care across Denmark.

### Setting

In Denmark, compulsory education is 10 years (0th to 9th grade). Hereafter, it is up to young people themselves to choose their further education. Two options are included in this research project: upper secondary schools and schools of health and social care. Upper secondary school is an academic youth education programme. It exists in four different forms (HTX, HHX, STX, and HF) which takes 2 to 3 years to complete. The common objective of upper secondary schools is to prepare young people for higher education (e.g. the university) [21]. School of health and social care is a vocational education. It educates social and health care assistants, social and health care workers, and pedagogical assistants over a period of 2 to 4 years. The common objective of schools of health and social care is to educate students on how to take care of people who cannot fend for themselves [22].

### Eligibility criteria

We included Danish upper secondary schools and schools of health and social care with no exclusion criteria. To be able to participate, consent from headmasters/headmistresses was essential. Each participating school was allowed to include between 1 and 4 teachers for teacher training. One of the inclusion criteria for students was an age of 16 to 24 years. Students were able to opt out of the trial by not completing questionnaires.

### Intervention

**Fig. 1:**
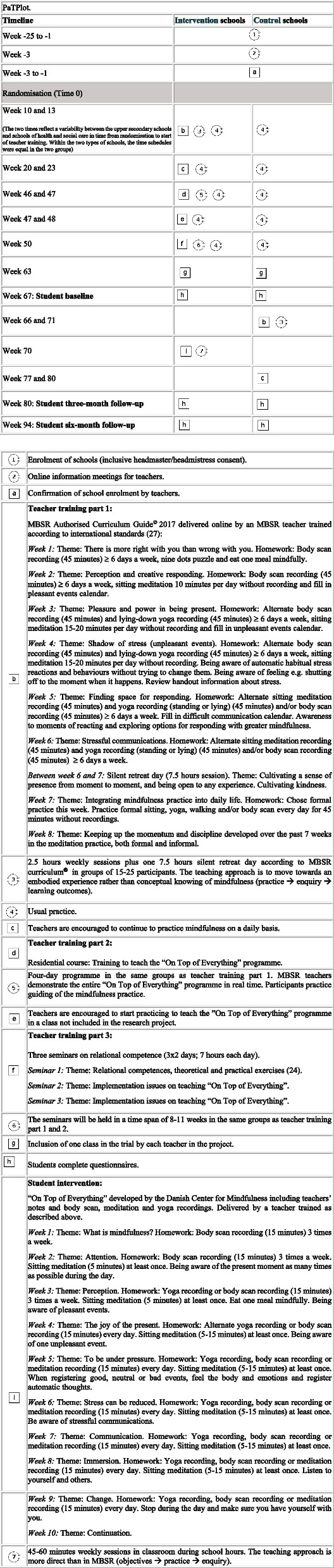
Timeline and description of the research activities and intervention contents in the project

The intervention is a multi-level, multi-component complex intervention. It consists of a teacher training programme and the mindfulness programme “On Top of Everything” delivered to students. Schools were randomised to begin teacher training in 2020 (intervention group) or in 2021 (control group). The timeline for research activities and intervention content is outlined in Fig. 1 [23]. The teacher training proceeds during a period of approximately 1 year and is facilitated by two MBSR instructors from the Danish Center for Mindfulness, Aarhus University (one with a professional background as an upper secondary school teacher and one as a psychologist).

The teacher training programme is based on three parts: First, participation in the MBSR programme, which is delivered in a combination of group-based face-to-face and live online teaching. The aim is to establish a formal mindfulness practice in the teachers. Second, completion of a 4-day residential course including training to teach the “On top of Everything” programme and access to teaching materials. Third, completion of three 2-day supervision seminars on implementation issues and relational competencies based on the work of The Danish Association for Promoting Life Wisdom in Children [24]. The teacher training programme includes information on potential adverse effects associated with mindfulness [25, 26].

The “On Top of Everything” programme consists of 10 well-described, weekly 45–60-min classroom sessions. Each session has a theme and teachers’ notes and body scan, meditation, and yoga recordings.

### Public and participant involvement

Teachers and students have been involved in the development of the “On Top of Everything” intervention through pilot tests.

### Outcome measures

Outcome measures are described in Table 1. Standard participant demographic information (gender, grade, and family setting) was collected at baseline. The outcome measures were collected at three time points (baseline, 3- and 6-month follow-ups). Information about mindfulness practice was collected at 3- and 6-month follow-ups in the intervention group. Our primary outcome is changes 6 months from baseline in the short version of the Warwick-Edinburgh Mental Well-Being Scale (SWEMWBS) [27] in the total population of students. A range of individual-level secondary outcome measures was chosen based on their ability to explore mental health.

**Table 1:** Description of the outcome measures assessed at baseline and 3 and 6 months among students

Measurement	Description
Demographic data	Data on age, gender, school, class, and family setting (only baseline)
Warwick-Edinburgh Mental Well-Being Scale. The short version (SWEMWBS) [27, 28]	The SWEMWBS is a seven-item questionnaire measuring mental well-being [27]. All items are positively worded. Participants are asked to rate each item on a 5-point Likert scale ranging from 1 (none of the time) to 5 (all the time) when considering the last two weeks. The overall score is calculated by summing the scores for each item. A higher score indicates a higher level of mental well-being. The scale is a valid and appropriate instrument to measure mental well-being in the Danish population [27]
Strengths and Difficulties Questionnaire. The youth self-report version (SDQ) [29]	The self-report version of the SDQ is a behavioural screening questionnaire measuring subjective well-being and social, emotional, and behavioural functioning among children and youth [29]. It consists of 25 statements, some positively and others negatively worded. Participants are asked to rate each item on a 3-point Likert scale ranging from 2 (very true) to 0 (not true). The 25 items are divided into five subscales of five items each, generating scores for emotional symptoms, conduct problems, hyperactivity/inattention, peer relationship problems, and prosocial behaviour. All subscales except the last one are summed to generate a total difficulties score. The subscales range from 0 to 10 with higher values indicating poorer well-being and functioning for four of the subscales (emotional symptoms, conduct problems, hyperactivity/inattention, and peer relationship problems) and better well-being and functioning for the subscale prosocial behaviour. The total difficulties score ranges from 0 to 40 with higher values indicating poorer well-being and functioning. Goodman et al. have shown that the odds ratio for having a mental disorder was 1.23 (95% CI 1.21 to 1.25) per one-point increase in the total difficulties score and that the odds ratio for developing a mental disorder within a 3-year period was 1.16 (95% CI 1.13 to 1.18) per one-point increase in the total difficulties score [30]. In 2019, Danish SDQ norms were published [31]
Depression Anxiety Stress Scale. The short form version (DASS) [32]	The DASS short-form version is a 21-item questionnaire consisting of three subscales measuring self-rated symptoms of depression, anxiety, and stress [32]. Each subscale consists of seven items. Participants are asked to rate each item on a 4-point Likert scale ranging from 0 (did not apply to me at all) to 3 (applied to me very much or most of the time) when considering the last week. For each subscale, the points are added together and multiplied by two. Thus, it is possible to score between 0 and 42 points on each subscale [33]
Perceived Stress Scale (PSS) [34, 35]	The PSS is a 10-item self-report measure of subjective stress [34]. It consists of 10 questions indicating how often participants have found their life unpredictable, uncontrollable, and overloaded in the past month. All items are scored on a 5-point Likert scale ranging from 0 (never) to 4 (very often) calculating a total sum score between 0 and 40 with higher scores indicating higher levels of stress. The instrument has demonstrated good validity and reliability [35]
Brief Resilience Scale (BRS) [36, 37]	The BRS is a measure of resilience [36]. It consists of six statements, three positively and three negatively worded. Each item is scored on a 5-point Likert scale ranging from 1 (strongly disagree) to 5 (strongly agree). The points are added together and divided by five, calculating a total score with higher values indicating a greater ability to bounce back when experiencing adversity. The following cut-off points have been suggested. Scores from 1.00 to 2.99: low resilience. Scores from 3.00 to 4.30: normal resilience. Scores from 4.31 to 5.00: high resilience. Windle et al. have proposed BRS to be one of the most valid instruments to measure resilience [37]
Experiences Questionnaire (EQ). The decentering subscale [38]	The EQ decentering subscale is a validated 11-item self-report measure of decentering [38]. Decentering refers to the ability to observe thoughts and feelings as temporary and automatic events in the mind, rather than facts or true descriptions of reality. The items of the decentering factor assess three facets: the ability to distinguish one’s self from one’s thoughts, the ability not to automatically react to one’s negative experiences, and the capacity for self-compassion. All items are scored on a 5-point Likert scale ranging from 1 (never) to 5 (always). The total sum score ranges from 11 to 55, with higher scores indicating greater decentering [38]
EQ-5D-Y [39]	EQ-5D is a valid instrument to measure health-related quality of life (HRQoL) for use in economic evaluations. It comprises five dimensions: mobility, self-care, usual activities, pain or discomfort, and anxiety or depression. Furthermore, participants are asked to rate their overall health on the EQ VAS, a vertical scale from 0 (the worst health you can imagine) to 100 (the best health you can imagine)
Three-Item Loneliness Scale (T-ILS) [40]	The T-ILS is a short questionnaire measuring self-reported loneliness [40]. T-ILS consists of three items and is a shortened version of the UCLA Loneliness Scale consisting of 20 items [41]. Participants are asked to answer each question on a 3-point Likert scale ranging from 1 (hardly ever) to 3 (often). The points are added together calculating a total score ranging from three to nine with higher scores indicating a greater extent of loneliness [40]
Karolinska Sleep Questionnaire. Modified version [42]	Sleep problems during the past 4 weeks will be assessed with six items from a modified version of the Karolinska Sleep Questionnaire [42]. The items are responded to on a 5-point Likert scale ranging from 1 (all the time) to 5 (at no time)
Mindfulness practice (those allocated to intervention)	We will use questions on adherence which have been used in former research on mindfulness among children and adolescents [43]

### Sample size

We assume a 1-point increase between-group effect in SWEMWBS to be of public health relevance [44, 45]. From another study that analysed the intervention effect on SWEMWBS in a mixed model with three repeated measurements, and where cluster levels of teacher and school were taken into account, we found a within-person SD = 2.4. To detect an effect on 1 score point in SWEMWBS change, SD = 3.5 = SQRT (2) × 2.4 with 80% power, will require 194 participants in each group, 388 participants in total.

### Recruitment

We recruited upper secondary schools and schools of health and social care through emails to headmasters/headmistresses, online information meetings, and advertisements on our webpage (www.mindfulness.au.dk) and social media between May 2019 and October 2019. We informed the headmasters/headmistresses as well as interested teachers that schools would be randomised to begin teacher training in 2020 or in 2021. Furthermore, it was obligatory for each teacher to include one class with 15 to 30 students. Due to the logistics of teacher planning, it was not possible to include students before the randomisation of schools.

### Randomisation

#### Sequence generation

The schools were allocated into intervention or control schools in two runs: one for each type of school. Each school received a random number, and then the schools were sorted according to the number of teachers included in the project (1 or 2–4) and the random number. The schools on the list were then alternately allocated to intervention or control starting with a control. This procedure was used to try to balance the two arms according to the teachers included in the project. The allocation was performed using Microsoft Excel.

#### Allocation concealment mechanism

The allocation was performed by the third author, who was not involved in the recruitment, implementation, or data collection. The third author received a list of the schools with an anonymous ID concealing the true identity of the schools and whether or not each school had one or more teachers included in the project. The school were then allocated to control or intervention, as described above. After this, the third author received a list with the true identities of the schools; these were then merged onto the schools before the allocations were sent to the second author.

The characteristics of the schools are shown in Table 2.

**Table 2:** Characteristics of included schools in the project

Characteristics	Upper secondary schools		Schools of health and social care	
	Intervention schools, *n* = 15	Control schools, *n* = 15	Intervention schools, *n* = 6	Control schools, *n* = 7
Total number of teachers	21	23	15	17
Number of teachers				
1 teacher	12	9	1	2
2 teachers	1	4	2	1
3 teachers	1	2	2	3
4 teachers	1	0	1	1

### Blinding

Neither intervention providers nor study participants were blinded.

### Data collection and management

Quantitative data were collected and stored using the Research Electronic Data Capture (REDCap) tool hosted by Aarhus University [46]. The included teachers were asked to register the class recruited for the research project, to provide students (including absentees) with online access to the three questionnaires (baseline, 3- and 6-month follow-up), and to arrange a time for students to complete the questionnaires during school hours. Teachers and students received two reminders in case of non-respondence.

We informed the students about the trial and the option to opt out and not completing the questionnaires. Data collection procedures have been pilot-tested and improved several times. Reasons for teachers to opt out of the trial were registered continuously. Furthermore, we registered participation in the teacher training programme and also the teaching of the mindfulness programme. The teachers were asked to report adverse events through their teaching period. Teachers have access to supervision from psychologists and psychiatrists to ensure prevent harm and to take good care of participants who may suffer harm from trial participation.

After implementing the “On Top of Everything” programme, teachers in the intervention group were invited to participate in focus group interviews. The teachers were asked about their experience of implementing mindfulness in their schools and about which facilitators and barriers they encountered through the process.

### Statistical methods

Quantitative data will be analysed according to the intention-to-treat principle using a repeated measurement model with the systematic effect: gender, age, class, time (three time points), intervention, interaction between time and intervention, and random effect of school, teacher/class within school, and student within class. Confidence intervals and standard errors will be found by bootstrapping to adjust for possible deviation from the normality of the random effects. Exploring subgroup analysis will be conducted on gender, a vulnerable subgroup (the 15% with the lowest SWEMWBS score), and type of school. To take account of missing data, we will perform four sensitivity analyses representing scenarios with data not missing at random. Missing outcomes will be substituted with the model-based prediction adding or subtracting 0.02 SD in the intervention or control arm.

Qualitative data will be transcribed and analysed based on qualitative content analysis. Each focus group interview will be read and notes will be taken, and themes will be introduced, organised, and related to quotes that are open to interpretation and that continue to an analytic investigation and connection of themes and quotes from all focus group interviews.

#### Significance and potential impact of the project

This trial will estimate the effectiveness of a population-based strategy on mental health in Danish young people enrolled in education. It will investigate the effectiveness of a mindfulness-based intervention based on the well-described MBSR programme modified to young people by the Danish Center for Mindfulness, Aarhus University. Furthermore, it will investigate facilitators and barriers among teachers to implement mindfulness in schools.

### Trial status

In total, 43 schools and 76 teachers consented to participate in the trial (44 teachers from 30 upper secondary schools and 32 teachers from 13 schools of health and social care). Teachers have recruited approximately 1100 students. Three focus group interviews were conducted among teachers in the intervention group after the implementation of the “On Top of Everything” programme.

Teaching of the “On Top of Everything” programme among students in the intervention group took place during the spring semester of 2021. Interviews among teachers took place in June 2021. The last quantitative data (6-month follow-up) were collected among students in the autumn of 2021. We will start cleaning and analysing the data at the beginning of 2023.

### Dissemination of knowledge

The results will be submitted for publication in international peer-reviewed journals. The results will also be presented at national and international conferences and scientific meetings. We also attempt to communicate the results in the press and specialist journals, and we will communicate the results at webpages, LinkedIn profiles, and social media (Facebook and Instagram).

## Data Availability

The dataset will be available from the second author by reasonable request after publication.

## Abbreviations

BRS: Brief Resilience Scale
DASS: Depression Anxiety Stress Scale
EQ: Experiences Questionnaire
HRQoL: Health-related quality of life
MBSR: Mindfulness-based stress reduction
PSS: Perceived Stress Scale
REDCap: Research Electronic Data Capture
SDQ: Strengths and Difficulties Questionnaire
T-ILS: Three-Item Loneliness Scale
SWEMWBS: Warwick-Edinburgh Mental Well-Being Scale: the short version
WHO: World Health Organization
WHO-5: 5-Item World Health Organization Well-Being Index
